# Conservative therapy versus arthroscopic surgery of femoroacetabular impingement syndrome (FAI): a systematic review and meta-analysis

**DOI:** 10.1186/s13018-022-03187-1

**Published:** 2022-06-03

**Authors:** Yanlin Zhu, Peng Su, Tianhao Xu, Lei Zhang, Weili Fu

**Affiliations:** Department of Orthopedics, Orthopedic Research Institute, West China Hospital, Sichuan University, Chengdu, 610041 Sichuan Province China

**Keywords:** Femoroacetabular impingement syndrome, Arthroscopy, Conservative treatment

## Abstract

**Purpose:**

FAI (femoroacetabular impingement syndrome) is a common cause of hip pain, resulting in a decreased life quality. This study aims to compare the postoperative clinical outcome between arthroscopic surgery (AT) and conservative treatment (CT).

**Method:**

The six studies were selected from PubMed, Embase and OVID database. The data were extracted and analyzed by RevMan5.3. Mean differences and 95% confidence intervals were calculated. RevMan5.3 was used to assess the risk of bias.

**Result:**

Six observational studies were assessed. The methodological quality of the trials indicated five of six studies had a low risk of bias and one article had a high risk of bias. The differences were statistically significant between AT and CT for HOS (follow-up for 6 months), iHOT-33 (follow-up for 6 months) improvement, iHOT-33 (follow-up for 12 months) improvement, iHOT-33 (follow-up for 12 months), EQ-5D-5L index score (follow-up for 12 months) and AT showed higher benefits than CT. Meanwhile no statistically significant were found in iHOT-33 (follow-up for 6 months), EQ-5D-5L index score (follow-up for 6 months), EQ5D-VAS (follow-up for 6 months) and EQ5D-VAS (follow-up for 12 months).

**Conclusion:**

AT and CT both can have clinical effects when facing FAI. In our meta-analysis, hip arthroscopy is statistically superior to conservative treatment in both long-term and short-term effects.

## Introduction

FAI (femoroacetabular impingement syndrome) is a common cause of hip pain, resulting in a decreased life quality [1, 2]. It was first described as FAI in 2001 which Ganz et al. describe it as irregularities in femoral and acetabular anatomy [3, 4]. Subsequently, this concept was updated by the 2016 Warwick Agreement consensus statement as “a motion-related clinical diagnosis of the hip that represents symptomatic contact between the proximal femur and the acetabulum” [5]. Classified by pathology, there are three morphologies of FAI: (1) cam-type morphology with an aspherical femoral head; (2) pincer-type morphology with an over coverage of the femoral head; and (3) mixed-type morphology which has both pincer and cam type, is the most common morphology. Because of the abnormal contact between femur and acetabulum, it will cause focal cartilage defects, cartilage delamination, chondrolabral separation and labral tears, eventually leading to hip osteoarthritis [6–8].

There are multiple treatments for FAI at present, including conservative care and arthroscopic surgery. But there are limited indications to choose either of one as a primary treatment. Although Peters et al. found three criteria as an indication for surgery, some research still remains conservative care as the first step in addressing FAI due to a modest chance of improvement with a low risk of harm [9–11]. Conservative care does have a positive effect in treating FAI, Per Hölmich et al. found physical therapy treatment reaches a significant improvement in α angle and Tönnis grade at a mean follow-up of 8 years. Meanwhile, a higher number of articles also showed a good prognosis after arthroscopic surgery. As an example, N V Bardakos et al. set up a research and a higher median postoperative modified Harris hip score was observed in the arthroscopic group compared with the control group [12].

High-quality evidence from randomized controlled trials that investigate physical therapy compared with arthroscopic surgery for patients with FAI is lacking [13, 14]. It is difficult for clinical surgeons to make a medical decision based on insufficient evidence. The purpose of this systematic review and meta-analysis was to synthesize data using the available clinical evidence to compare the efficacy and outcomes of patients with FAI syndrome treated with hip arthroscopy versus those treated with conservative care alone.

## Method

### Inclusion and exclusion criteria

Articles that meet the following inclusion criteria are included: (1) published randomized controlled trials (RCTs) comparing arthroscopic surgery versus conservative care (e.g., physiotherapy) in the management of FAI syndrome; (2) clinical manifestations and imaging diagnosis of FAI syndrome; (3) a minimum 6-month clinical follow-up period; and (4) patient aged more than 18 years old. The exclusion criteria were: (1) the patient has a primary hip disease such as hip osteoarthritis; (2) the RCTs are not rigorous; (3) the studies did not report the clinical outcomes; and (4) all other types of research is excluded such as cohort studies, case–control studies, case series, individual case studies and unpublished abstract. Overall, patients included in this review were individuals aged 18 years or older with clinical and imaging diagnoses of FAI.

## Outcome

The primary outcome of interest was the International Hip Outcome Tool 33 (iHOT-33), both improvement data and primary data [15]. This outcome was designed to measure the hip-related quality of life in young adults with non-arthritic hip pain. The score ranges from 0 to 100, and a higher score indicates better functionality.

Secondary outcomes were included Hip Outcome Score (HOS) [16], Degree of Improvement on Hip VAS Pain Score [17], EQ-5D-5L index score and VAS score [18]. Other information included complications and adverse events in both treatment groups. Arthroscopic surgery details such as the surgery approach and different types of morphology were collected. The number of people lost to follow-up was also recorded as the study characteristic.

## Search strategy

We used a text search strategy using the ("femoracetabular" [MeSH term] OR "femoro-acetabular" OR "femoro acetabular") AND (impingement [MeSH term] OR "impingement syndrome"). Specifically, we searched the PubMed, Embase and OVID database from inception to September 20, 2021. We also assessed the bibliographies of identified studies to seek additional articles. We did not add language restrictions.

## Study selection

A single reviewer screened all citations and abstracts generated by the literature search and applied the selection criteria. Identified randomized trials were assessed for inclusion by 2 reviewers. Any disagreement between them on the eligibility of certain studies was resolved through discussion with a third reviewer. The titles of journals and names of authors were not masked during the study selection process.

## Quality assessment

The risk of bias graph in Review Manager 5.3 was used to evaluate the methodological quality of included studies in this meta-analysis. This 7-element checklist qualitatively assesses various aspects of trial quality (random sequence generation, allocation concealment, blinding of participant and personnel, blinding of outcome assessment, incomplete outcome data, selective reporting and other bias) using an ordinal scoring system comprising high risk, low risk or unclear risk response options for each statement on the Review Manager 5.3. A higher score obtained with Review Manager 5.3 is indicative of higher methodological study quality. We did not perform the assessment of publication bias with a funnel chart because we had less than ten studies for each comparison in this review.

## Statistical analysis

Data were extracted independently from included studies on data abstraction forms by a single reviewer. We extracted data on participants’ characteristics (sex, age, duration of symptoms, severity of the condition at the beginning of the study), treatment dosage (number of sessions, duration of each treatment session, etc.) and description of the intervention. In addition, further data were retrieved if the authors deceit med necessary. Reports on study funding and registration in some prospective databases were also extracted.

Meta-analysis was planned if studies were clinically homogeneous in population, intervention and outcome assessment and we used Review Manager 5 for all analyses. As all outcomes that can analyze were continuous results, we quantified the treatment effects with the mean difference (MD), effect sizes and 95% confidence intervals (CI), and *P* < 0.05 was considered statistically significant for all outcome measures. A fixed-effects model was used to pool results from comparable studies in the absence of significant heterogeneity (*I*^2^ < 60%), whereas a random-effects model was used to pool results when significant heterogeneity was present (*I*^2^ > 60%).

## Result

### Studies identification and inclusion

**Fig. 1:**
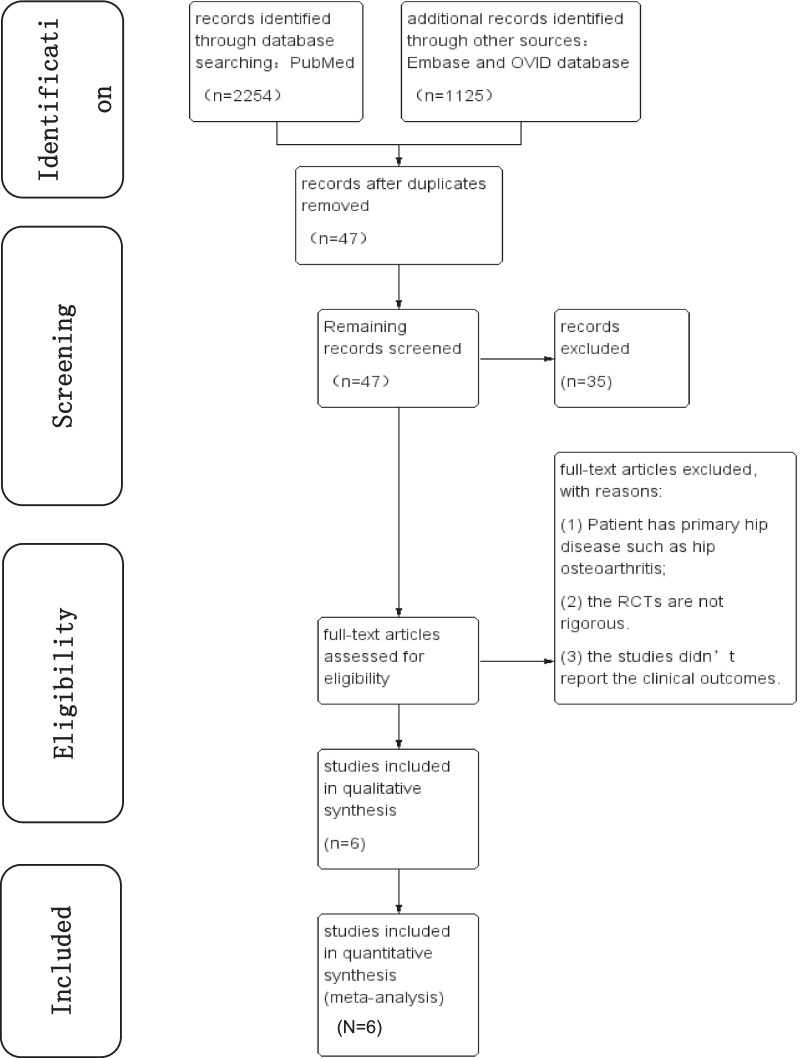
Search strategy results (RCTs, randomized controlled trials.)

We conducted article searches in the PubMed, Embase and OVID and other databases and our search strategy create 3379 titles that were eligible for the review. After removing duplicate articles, 47 articles remain. Based on carefully screening the remained articles’ titles and abstracts, 35 irrelevant articles of them were excluded. After applying the inclusion criteria, six randomized controlled trials were excluded: 3 articles included patients older than 60 years and did not specify whether they had osteoarthritis, 2 articles lacked reports of loss to follow-up and adverse events, and 1 article had flaws in randomization concealment and blinding. Finally, 6 studies were included in this systematic review and meta-analysis. The detail of the selection process is listed in Fig. 1.

## Study characteristics

In summary, six-level RCTs were included in this systematic review and meta-analysis [19–24]. The including studies were conducted in three countries (UK, USA and Australia) and involved 1187 patients (598 patients in conservative treatment group and 589 patients in arthroscopic surgery group) aged from 29.7 to 49.6 years. Among 1187 patients, 550 were female and 637 were male. The follow-up time ranges from 8 to 24 months. Weight, height and BMI were reported in some articles and did not present in others. The clinical outcomes of the studies were evaluated mainly based on the pooled results of iHOT-33, mHHS, visual analog scale, HOS-ADL, NAHS, Lower Extremity Function Score, dGEMRIC, HOOS, SF-12, PCS, MCS, EQ-5D. Adverse events were available in most of the studies, but none of them report severe events. A total of 64 people were lost to follow-up in the included literature, including 10 who did not receive treatment, 5 who could not be contacted, 34 who could not receive continuous treatment and 5 who were not eligible for inclusion. The details of including study characteristics are summarized in Table 1 and Table 1 continued.

**Table 1:** Study characteristics

Title	No. of patients		Age		Gender (female:male)		Height (cm)		Weight (kg)		BMI (kg/m2)	
	CT	AT	CT	AT	CT	AT	CT	AT	CT	AT	CT	AT
Realpe, A.2021	177	171	N	N	64:113	71:100	N	N	N	N	N	N
Martin, S.2021	44	46	49.1	49.6	20:24	23:23	N	N	N	N	26.8	27.1
Hunter, D.2021	50	49	32.9	32.9	26:24	31:18	N	N	N	N	N	N
Palmer, A. 2018	110	112	36.0	36.4	73:37	74:38	171.9	170.5	78.6	76.1	26.6	25.9
Mansell, N.2018	40	40	30.6	29.7	14:26	19:21	N	N	N	N	27.47	28.23
Griffin, D.2018	177	171	35.2	35,4	64:113	71:100	N	N	N	N	N	N

Title	Follow-up (month)		Outcome	Adverse event		Morphology	
	CT	AT		CT	AT	CT	AT
Realpe, A.2021	12	12	iHOT-33, questionnaire	N	N	N	N
Martin, S.2021	12	12	iHOT-33, mHHS, visual analog scale, HOS-ADL, NAHS, Lower Extremity Function Score	N	N	N	N
Hunter, D.2021	12	12	dGEMRIC, iHOT-33, HOOS, SF-12, PCS, MCS, EQ-5D	muscle soreness	numbness in the groin, leg or foot	Pincer: 9, Cam: 30, Mix: 10	Pincer: 9, Cam: 32, Mix: 9
Palmer, A. 2018	8	8	HOS-ADL, HOS sport subscale, NAHS, HAGOS, OHS, iHOT-33	chronic pain	Superficial wound Infection, Injury to the lateral cutaneous nerve	Pincer: 0, Cam: 104, Mix: 6	Pincer: 1, Cam: 104, Mix: 7
Mansell, N.2018	24	24	HOS, iHOT-33, Global Rating of Change	N	N	N	N
Griffin, D.2018	12	12	iHOT-33, EQ-5D-5L, SF-12	Muscle soreness, Numbness in the groin, leg or foot, hip pain or stiffness,	Muscle soreness, Numbness in the groin, leg or foot, hip pain or stiffness, superficial wound, hip joint infection	Pincer: 14, Cam: 133, Mix:30	Pincer: 13, Cam: 129, Mix:29

*CT* Conservative treatment: Including but not limited to patient education, activity modifications, oral anti-inflammatories, physical therapy and intra-articular musculoskeletal injection therapies. *AT* Arthroscopic surgery N: Not present dGEMRIC: delayed gadolinium-enhanced magnetic resonance imaging (MRI) of cartilage. iHOT-33: International Hip Outcome Tool HOOS: the Hip disability and Osteoarthritis Outcome Score, SF-12 12-item Short Form Health Survey. *PCS* Physical component score. *MCS* Mental component score HOS-ADL: activities of daily living subscale of the hip outcome score *HOS* Hip outcome score *NAHS* Non-arthritic hip score, *HAGOS* Copenhagen hip and groin outcome score *OHS* Oxford hip score, *mHHS* Modified Harris Hip Score

## Methodological assessment of study quality

**Fig. 2:**
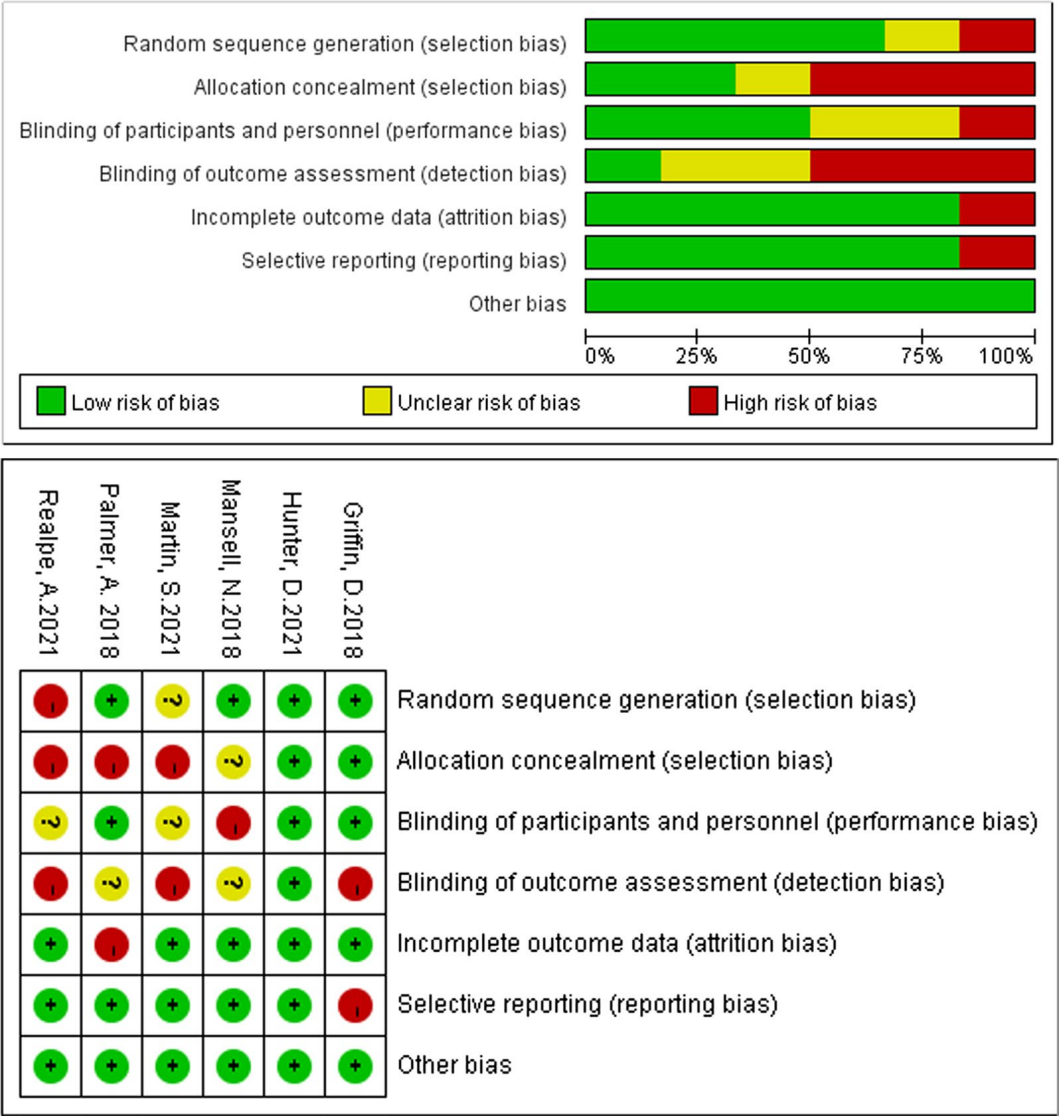
Risk of bias used Review Manager 5.3

We use Review Manager 5.3 to operate an assessment of the including six articles. The detailed results were present in Fig. 2. Six items were used to assess the risk of bias [25]. In particular, most of the studies get a high evaluation in both attrition bias and reporting bias. Overall, five of six studies were high quality and had a low risk of bias. Only one article was low quality and had a high risk of bias.

## Outcomes

Comparison of Hip Outcome Score between AT and CT (follow-up for 6 months).

**Fig. 3:**
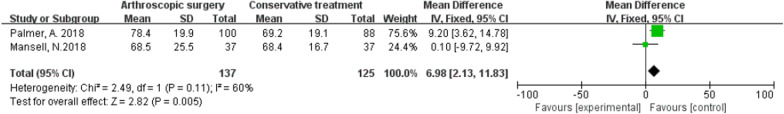
Forest plot of comparison: postoperative Hip Outcome Score (follow-up for 6 months) between AT and CT

Comparison of postoperative Hip Outcome Score (follow-up for 6 months) between AT and CT was conducted among the 2 studies [22, 23], which included 262 patients (137 patients receiving AT and 125 patients receiving CT), the detailed information is shown in Fig. 3. Heterogeneity testing showed that there was a little high heterogeneity among the studies (*P* = 0.11, *I*^2^ = 60%); following the method described before, the fixed-effects model was used to analyze the data from 2 studies. The pooled result showed that the difference was statistically significant between the AT group and the CT group (MD = 6.98 CI = 2.13 to 11.83 *Z* = 2.82 *P* = 0.0005 < 0.05).

Comparison of International Hip Outcome Tool–33 Questions (follow-up for 6 months) improvement.

**Fig. 4:**
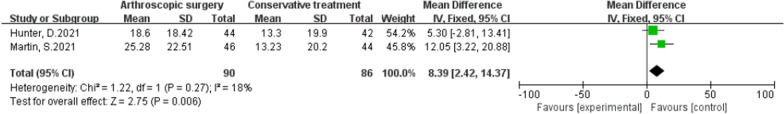
Forest plot of comparison: postoperative International Hip Outcome Tool–33 Questions (follow-up for 6 months) improvement between AT and CT

Comparison of postoperative International Hip Outcome Tool–33 Questions (follow-up for 6 months) improvement between AT and CT was conducted among the 2 studies [20, 21], which included 176 patients (90 patients receiving AT and 86 patients receiving CT), the detailed information is shown in Fig. 4. The postoperative improvement data are based on each article’s own baseline data. Heterogeneity testing showed that there was a low heterogeneity among the studies (*P* = 0.27, *I*^2^ = 18%); following the method described before, the fixed-effects model was used to analyze the data from 2 studies. The pooled result showed that the difference was statistically significant between the AT group and the CT group (MD = 8.39 CI = 2.42 to 14.37 *Z* = 2.75 *P* = 0.005 < 0.05).

Comparison of International Hip Outcome Tool–33 Questions (follow-up for 6 months).

**Fig. 5:**
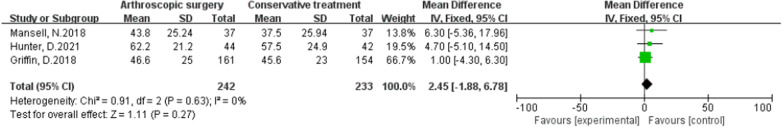
Forest plot of comparison: postoperative International Hip Outcome Tool–33 Questions (follow-up for 6 months) between AT and CT

Comparison of postoperative International Hip Outcome Tool–33 Questions (follow-up for 6 months) between AT and CT were conducted among the 3 studies [21, 23, 24], which included 475 patients (242 patients receiving AT and 233 patients receiving CT), the detailed information is shown in Fig. 5. Heterogeneity testing showed that there was no heterogeneity among the studies (*P* = 0.63, *I*^2^ = 0%); following the method described before, the fixed-effects model was used to analyze the data from 3 studies. The pooled result showed that the difference was not statistically significant between the AT group and the CT group (MD = 2.45 CI = − 1.88 to 6.78 *Z* = 1.11 *P* = 0.27 > 0.05).

Comparison of International Hip Outcome Tool–33 Questions (follow-up for 12 months) improvement.

**Fig. 6:**
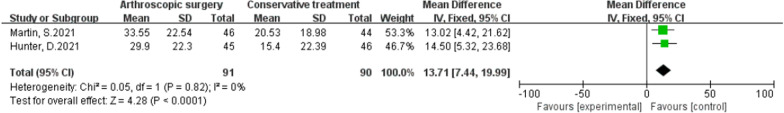
Forest plot of comparison: postoperative International Hip Outcome Tool–33 Questions (follow-up for 12 months) improvement between AT and CT

Comparison of postoperative International Hip Outcome Tool–33 Questions (follow-up for 12 months) improvement between AT and CT was conducted among the 2 studies [20, 21], which included 181 patients (91 patients receiving AT and 90 patients receiving CT), the detailed information is shown in Fig. 6. Heterogeneity testing showed that there was no heterogeneity among the studies (*P* = 0.82, *I*^2^ = 0%); following the method described before, fixed-effects model was used to analyze the data from 3 studies. The pooled result showed that the difference was statistically significant between the AT group and the CT group (MD = 13.71 CI = 7.44 to 19.9 Z = 4.28 *P* < 0.0001 < 0.05).

Comparison of International Hip Outcome Tool–33 Questions (follow-up for 12 months).

**Fig. 7:**
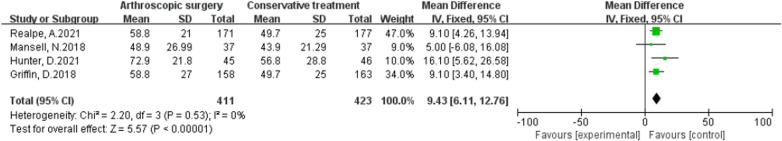
Forest plot of comparison: postoperative International Hip Outcome Tool–33 Questions (follow-up for 12 months) between AT and CT

Comparison of postoperative International Hip Outcome Tool–33 Questions (follow-up for 12 months) between AT and CT were conducted among the 4 studies [19, 21, 23, 24], which included 834 patients (411 patients receiving AT and 423 patients receiving CT), the detailed information is shown in Fig. 7. Heterogeneity testing showed that there was no heterogeneity among the studies (*P* = 0.53, *I*^2^ = 0%); following the method described before, fixed-effects model was used to analyze the data from 4 studies. The pooled result showed that the difference was statistically significant between the AT group and the CT group (MD = 9.43 CI = 6.11 to 12.76 *Z* = 5.57 *P* < 0.0001 < 0.05).

Comparison of EQ-5D-5L index score (follow-up for 6 months).

**Fig. 8:**
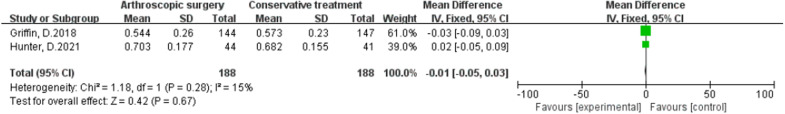
Forest plot of comparison: postoperative EQ-5D-5L index score (follow-up for 6 months) between AT and CT

Comparison of postoperative EQ-5D-5L index score (follow-up for 6 months) between AT and CT was conducted among the 2 studies [21, 24], which included 376 patients (188 patients receiving AT and 188 patients receiving CT), the detailed information is shown in Fig. 8. A low heterogeneity was found among the studies ( *p* = 0.28 *I*^2^ = 15%); following the method described before, fixed-effects model was used to analyze the data from 2 studies. The pooled result showed that the difference was no statistically significant between the AT group and the CT group (MD = − 0.01 CI = − 0.05 to 0.03 *Z* = 0.42 *P* = 0.67 > 0.05).

Comparison of EQ-5D-5L index score (follow-up for 12 months).

**Fig. 9:**
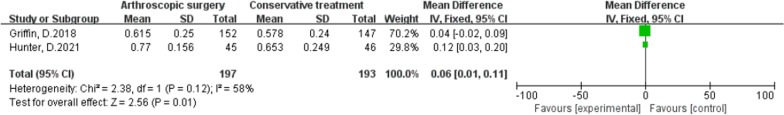
Forest plot of comparison: postoperative EQ-5D-5L index score (follow-up for 12 months) between AT and CT

Comparison of postoperative EQ-5D-5L index score (follow-up for 12 months) between AT and CT was conducted among the 2 studies [21, 24], which included 390 patients (197 patients receiving AT and 193 patients receiving CT), the detailed information is shown in Fig. 9. A little high heterogeneity was found among the studies ( *p* = 0.28 *I*^2^ = 58%); following the method described before, the fixed-effects model was used to analyze the data from 2 studies. The pooled result showed that the difference was statistically significant between the AT group and the CT group (MD = 0.06 CI = 0.01 to 0.11 *Z* = 2.56 *P* = 0.01 < 0.05).

Comparison of EQ5D-VAS (follow-up for 6 months).

**Fig. 10:**
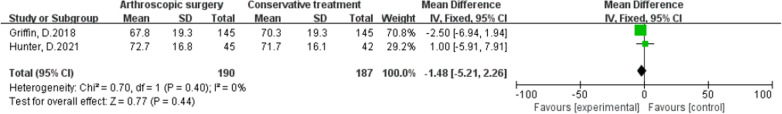
Forest plot of comparison: postoperative EQ5D-VAS (follow-up for 6 months) between AT and CT

Comparison of postoperative EQ5D-VAS (follow-up for 6 months) between AT and CT was conducted among the 2 studies [21, 24], which included 377 patients (190 patients receiving AT and 187 patients receiving CT), the detailed information is shown in Fig. 10. No heterogeneity was found among the studies ( *p* = 0.40 *I*^2^ = 0%); following the method described before, the fixed-effects model was used to analyze the data from 2 studies. The pooled result showed that the difference was not statistically significant between the AT group and the CT group (MD = − 1.48 CI = − 5.21 to 2.26 *Z* = 0.77 *P* = 0.44 > 0.05).

Comparison of EQ5D-VAS (follow-up for 12 months).

**Fig. 11:**
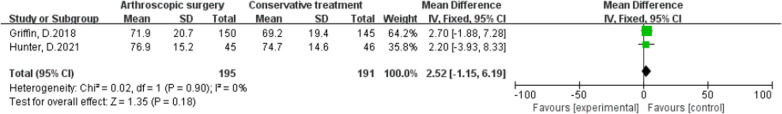
Forest plot of comparison: postoperative EQ5D-VAS (follow-up for 12 months) between AT and CT

Comparison of postoperative EQ5D-VAS (follow-up for 12 months) between AT and CT was conducted among the 2 studies [21, 24], which included 377 patients (190 patients receiving AT and 187 patients receiving CT), the detailed information is shown in Fig. 11. No heterogeneity was found among the studies ( *p* = 0.90 *I*^2^ = 0%); following the method described before, the fixed-effects model was used to analyze the data from 2 studies. The pooled result showed that the difference was not statistically significant between the AT group and the CT group (MD = 2.52 CI = − 1.15 to 6.19 *Z* = 1.35 *P* = 0.18 > 0.05).

## Conclusion

In our study, we identified six RCTs studies for investigating the clinical outcomes of AT versus CT, and we collect adverse events and each morphology. In our meta-analysis, the result showed that the differences were statistically significant between AT and CT for HOS (follow-up for 6 months), iHOT-33 (follow-up for 6 months) improvement, iHOT-33 (follow-up for 12 months) improvement, iHOT-33 (follow-up for 12 months), EQ-5D-5L index score (follow-up for 12 months) and AT showed higher benefits than CT. Meanwhile no statistically significant were found in iHOT-33 (follow-up for 6 months), EQ-5D-5L index score (follow-up for 6 months), EQ5D-VAS (follow-up for 6 months) and EQ5D-VAS (follow-up for 12 months).

High heterogeneity was found in comparison of HOS (follow-up for 6 months) and Comparison of EQ-5D-5L index score (follow-up for 12 months), other studies were present with low or no heterogeneity. High heterogeneity may be related to the design, interventions and research methods of each study [26]. Different regions and different races may have different pathological prognoses [27]. So the association between different risk factors and prognosis cannot be described. We look forward to more relevant research with an exhaustive baseline and outcomes in the future.

In terms of individual results of each study, David, Palmer, Griffin and Martin’s studies appear a preference that AT achieve superior outcomes compared with CT [20–22, 24]. While Mansell et al. found the clinical outcomes of AT are no better than CT [23]. Moreover, the study conducted by Realper indicated that two treatments each have its own merits [19]. Due to the RCTs, we can collect now, both AT and CT can reach the clinical benefit change. But there is no consistent conclusion about which treatment is more beneficial [28]. Based on our meta-analysis, AT showed statistically significant in HOS (follow-up for 6 months), iHOT-33 (follow-up for 6 months) improvement, iHOT-33 (follow-up for 12 months) improvement, iHOT-33 (follow-up for 12 months), EQ-5D-5L index score (follow-up for 12 months), both in sensory function and motor function. Despite no statistically significant results were presented with iHOT-33 (follow-up for 6 months), EQ-5D-5L index score (follow-up for 6 months), EQ5D-VAS (follow-up for 6 months) and EQ5D-VAS (follow-up for 12 months). But in these scales, AT still reach the minimally clinically important difference and showed the clinical effect.

In particular, the iHOT-33 (follow-up for 6 months) improvement showed statistically significant yet iHOT-33 (follow-up for 6 months) presented no statistically significant. Their heterogeneities are all low (*I*^2^ = 18% and *I*^2^ = 0). Such a result may be due to the baseline of each study being different so that the improvement showed statistically significant, but the original data showed not. We can also interpret this special result as AT is better than CT in clinical outcomes because AT does have clinical effect and in the meantime, under the background of non-heterogeneous baseline data, AT has shown a more excellent improvement effect and these data are statistically significant. The statistically significant in iHOT-33 (follow-up for 12 months) improvement, iHOT-33 (follow-up for 12 months) and EQ-5D-5L index score (follow-up for 12 months) also support our argument and show that arthroscopic surgery has a better long-term prognosis effect.

Compared with recent related literature, an updated systematic review and meta-analysis by Bastos, R showed that surgical treatment is not superior to conservative treatment for femoroacetabular impingement syndrome in the short term, and there is low-quality evidence that it is not superior in the medium term [29]. The result is inconsistent with our results, but our study included more RCTs (six vs three) and we use more scale comparisons as clinical results. Another literature presented the same result as our result, though it only contained three studies and one outcome [30]. Based on the current lack of high-quality evidence, in contrast, our results are more convincing.

Schwabe MT conducted a meta-analysis that included three studies, based on the comparison of iHOT-33 and HOS-ADL, and concluded that the outcome of surgery is better than PT [31]. But compared to our article, we included six studies in our analysis, which is larger than the number of studies by Schwabe MT et al., and we have more outcome measures. With a larger sample size and more outcome indicators, the conclusions drawn are more meaningful for future clinical decisions. Moreover, in the article of Schwabe MT, the results of the two outcome indicators were consistent: surgical treatment was better than conservative treatment. However, after the follow-up was divided according to time points in our article, the IHOT-33 index showed no statistical difference at the 6-month follow-up time, but there was a statistical difference at the 12-month time point. We, therefore, present our conclusions.

In the article published by Dwyer T, similarly, only three articles were included [32]. Moreover, the statistical method of frequency weighting was used in the paper to unify the follow-up time of IHOT-33 to 10 months. Such comparisons can only yield results that surgical treatment is superior to conservative treatment. However, the disease is a dynamic process, and our article can conclude from the difference in follow-up time: surgical treatment can achieve better results faster than conservative treatment in a shorter period.

The article by Kim CH included 5 articles, including 3 RCTs, 1 prospective cohort study and 1 retrospective cohort study [33]. Therefore, in terms of methodological quality, it is inferior to the articles that are all included in RCTs, and the conclusions drawn are also slightly less scientific. Our article analyzed the outcome index EQ-5D-5L at both follow-up nodes, and the conclusions reached were consistent with our final conclusions, and based on this conclusion, we made recommendations for clinical treatment. Because only the results of IHOT-33 were statistically significant in Kim CH's article, only one possibility was raised in the conclusion "Further studies will be needed to conclusively determine if one strategy is superior to the other for treating FAI," and no recommendations for clinical treatment were made.

Our limitations of this study should be mentioned. The sample size is still small for a meta- analysis. It may cause high heterogeneity between different studies and incorrect results. Secondly, the data we extracted ignore different prognosis brought by different morphology types due to the based on unclassified raw data. It may create report bias. More large-sample, multicenter, high-quality RCTs are needed to verify the outcomes of this meta-analysis.

## Conclusion

AT and CT both can have clinical effects when face FAI. In our meta-analysis, hip arthroscopy is statistically superior to conservative treatment in both long-term and short-term effects. AT can achieve better results faster than CT in a shorter period of time. Arthroscopic treatment is more recommended in people who need better prognosis improvement in a shorter time. This may give a reasonable direction for future treatment.

## Data Availability

The datasets used or analysis during the current study are available from corresponding author on reasonable request.
